# Comparative Study of Remimazolam and Midazolam During Sedated Upper Gastrointestinal Endoscopy: A Propensity Score Matching Analysis

**DOI:** 10.1002/jgh3.70100

**Published:** 2025-01-20

**Authors:** Ryoji Ichijima, Hisatomo Ikehara, Tomomi Sugita, Daisuke Yamaguchi, Yasuhiko Nagata, Kanako Ogura, Mitsuru Esaki, Yosuke Minoda, Hiroyuki Ono, Yuki Maeda, Shinsuke Kiriyama, Tetsuya Sumiyoshi, Yuichi Kanmura

**Affiliations:** ^1^ Division of Gastroenterology and Hepatology, Department of Medicine Nihon University School of Medicine Tokyo Japan; ^2^ Department of Gastroenterology Kiriyama Clinic Takasaki Gunma Japan; ^3^ Department of Gastroenterology, Internal Medicine Kitasato University School of Medicine Sagamihara Japan; ^4^ Department of Gastroenterology National Hospital Organization Ureshino Medical Center Ureshino Japan; ^5^ Department of Gastroenterology Nagata Surgery and Gastroenterological Clinic Nishitokyo‐shi Tokyo Japan; ^6^ Department of Medicine and Bioregulatory Science, Graduate School of Medical Sciences Kyushu University Fukuoka Japan; ^7^ Division of Endoscopy Shizuoka Cancer Center Shizuoka Japan; ^8^ Department of Gastroenterology Tonan Hospital Hokkaido Japan; ^9^ Department of Anesthesiology Fujimoto General Hospital Miyazaki Japan

**Keywords:** endoscopy, gastrointestinal, midazolam, propensity score, remimazolam

## Abstract

**Aim:**

This study aimed to compare the use of remimazolam and midazolam in upper gastrointestinal endoscopy in Japan as a sub‐analysis of data from an investigator‐initiated clinical trial of remimazolam.

**Methods and Results:**

Patients in two groups were matched using propensity score matching. We evaluated the time from the end of the gastrointestinal endoscopy until discharge, the time from the end of the procedure until awakening, and adverse events. Overall, 36 participants from the clinical trial population who underwent upper gastrointestinal endoscopy using remimazolam and 199 patients who underwent the procedure with midazolam during the same period were included in this study. Following propensity score matching, 34 patients in both groups were matched. The median time from the end of the procedure until awakening was 27.0 min (23.0–40.5 min) in the midazolam group (Group M) and 0 min (0–5.0 min) in the remimazolam group (Group R); the median time from the end of the upper gastrointestinal endoscopy until discharge was 39.0 min (35.0–52.5 min) in Group M and 5.0 min (0–5.0 min) in Group R (*p* < 0.01). Reported adverse events were hypotension and hypoxemia in one patient in Group R.

**Conclusion:**

Compared with midazolam, remimazolam significantly shortened the time to patient awakening and duration until the patient could leave the endoscopy room.

**Trial Registration:** The main study (REM‐IICT JP1) is registered with the Japan Registry of Clinical Trails: jRCT2031200360

## 1 Introduction

Upper gastrointestinal endoscopy plays an important role in the early detection of malignant tumors but may cause pain and anxiety in patients. Hence, some patients prefer not to undergo endoscopy owing to fear or anxiety about the procedure [1]. Sedation makes endoscopy more comfortable for patients, alleviates anxiety, and reduces bodily movements during the procedure, facilitating a simpler and more efficient execution [2, 3]. Therefore, the demand for gastrointestinal endoscopy under sedation has increased in recent years [4, 5].

In Japan, only a few drugs have been approved for use as sedatives during gastrointestinal endoscopy, and the off‐label use of benzodiazepines is common [6]. Midazolam is the most frequently used benzodiazepine during gastrointestinal endoscopy in routine clinical practice [7, 8]. However, the sedative effect of midazolam is prolonged beyond the completion of the procedure, requiring some time before the patient can leave the endoscopy room [9]. When flumazenil was used as a benzodiazepine antagonist, the sedation level improved within 5 min after its administration; however, there is a risk of resedation, as flumazenil has a shorter half‐life than midazolam [10].

Remimazolam is a novel ultra‐short‐acting benzodiazepine approved by the US Food and Drug Administration in 2020. In Japan, it is only authorized for general anesthesia; however, its usefulness in gastrointestinal endoscopy has been widely reported overseas [11, 12, 13, 14, 15, 16, 17, 18, 19]. Because the half‐life of remimazolam is 45 min, which is much shorter than the 4 h half‐life of midazolam, its use should shorten the time until the patient can leave the endoscopy room after the procedure. Therefore, it may be a suitable drug for use in endoscopy that does not require long‐term sedation. In Japan, we previously conducted an investigator‐initiated trial of remimazolam in gastrointestinal endoscopy (REM‐IICT JP01 study) and reported its value [12, 13]. However, no study has compared the use of remimazolam and midazolam in upper gastrointestinal endoscopy in Japan. Therefore, we conducted a comparative investigation with midazolam as a post hoc analysis of the REM‐IICT JP01 study.

## 2 Methods

### 2.1 Study Design

This study was a post hoc analysis of the REM‐IICT JP01 study, which was registered as a clinical trial (jRCT2031200360). The REM‐IICTJP01 study of Japanese patients undergoing gastrointestinal endoscopy was divided into two steps: a dose‐finding step to determine the appropriate dose of remimazolam and a verification step to assess the efficacy and safety of remimazolam in comparison with placebo.

In this study, we performed a single‐center retrospective, comparative investigation of patients undergoing upper gastrointestinal endoscopy using remimazolam and midazolam at Nihon University Hospital during the same period.

**FIGURE 1:**
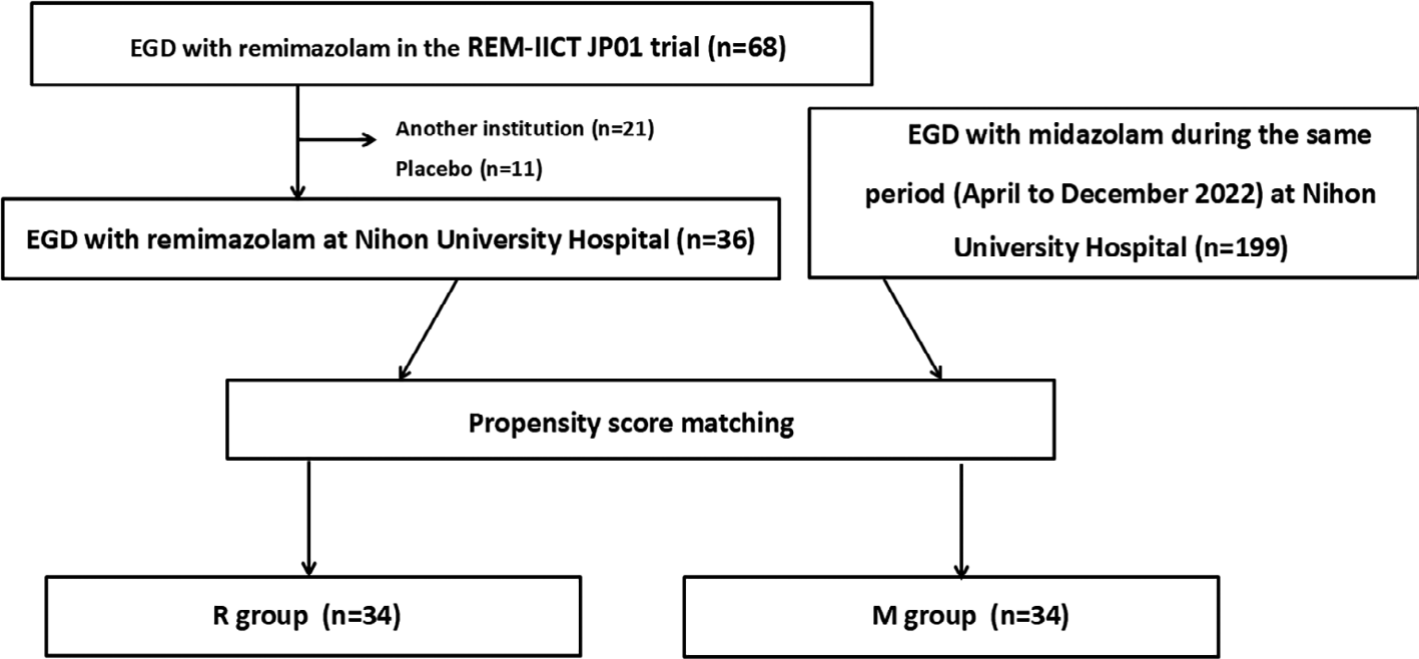
Study flow chart. EGD, upper gastrointestinal endoscopy; M group, midazolam group; R group, remimazolam group.

The remimazolam group comprised 36 of 68 patients from the REM‐IICT‐JP01 study, conducted between April and December 2022, who underwent upper gastrointestinal endoscopy under sedation with remimazolam. Eleven patients in the placebo group and 21 patients who underwent the procedure in other institutions were excluded. The midazolam group comprised 199 patients who underwent upper gastrointestinal endoscopy under sedation with midazolam and for whom sufficient information was available (Figure 1).

### 2.2 Ethical Approval

The current study was a post hoc analysis of a study (REM‐IICT JP01) and was approved by the Institutional Review Board of Nihon University Hospital. The main study (REM‐IICT JP01) protocol was approved by the Institutional Review Board of each participating institution and was registered in the Japan Registry of Clinical Trails (jRCT2031200360). This study was conducted in compliance with the principles of the Declaration of Helsinki. Written informed consent was obtained from all patients who underwent endoscopy.

### 2.3 Inclusion and Exclusion Criteria

The remimazolam group included patients who met the inclusion criteria of the clinical trial. The main inclusion criteria were as follows: (1) Japanese individuals scheduled to undergo upper gastrointestinal endoscopy without the use of any other sedative and (2) body mass index (BMI) < 30 kg/m^2^.

The main exclusion criteria were as follows: (1) severe respiratory disease, (2) Mallampati class III or above, (3) previous sleep apnea, (4) pregnant or lactating women or women with childbearing potential, and (5) considered by a doctor to be unsuitable for participating in the study for any other reason.

The midazolam group included all Japanese patients who had undergone upper gastrointestinal endoscopy under sedation with midazolam without the concomitant use of any other sedative in our hospital. Both groups targeted upper gastrointestinal endoscopy performed for the purpose of screening or surveillance, and excluded therapeutic endoscopy procedures such as hemostasis for gastrointestinal bleeding, ileus tube insertion, and endoscopic submucosal dissection. Upper gastrointestinal endoscopy was conducted without concomitant analgesics such as pethidine hydrochloride in both groups.

### 2.4 Study Procedure and Drug Administration

Patients fasted from 9 p.m. on the day before endoscopy. As a pretreatment for upper gastrointestinal endoscopy, lidocaine spray was used as necessary for pharyngeal anesthesia.

An initial dose of midazolam or remimazolam was gradually administered intravenously. After an interval of at least 2 min from the start of the initial administration, the sedation level was assessed using the Modified Observer's Assessment of Alertness/Sedation (MOAA/S) score [20] (Table 1), and gastrointestinal endoscopy was started after sedation (MOAA/S score ≥ 4) had been achieved. If assessment using the MOAA/S score after at least 2 min had elapsed since the start of initial administration showed that sedation had not been obtained, an additional dose was gradually administered if the MOAA/S score was 5. After an interval of at least 2 min from the start of additional administration, the sedation level was assessed using the MOAA/S score; if the MOAA/S score was 5, a further additional dose was gradually administered.

**TABLE 1:** Modified Observer's Alertness/Sedation (MOAA/S) scale.

MOAA/S scale	
5	Responds readily to name spoken in normal tone
4	Lethargic response to name spoken in normal tone
3	Responds only after name is called loudly and/or repeatedly
2	Responds only after mildly prodding or shaking
1	Responds only after painful trapezius squeeze
0	No response after painful trapezius squeeze

After initiation, the procedure was performed using either GIF‐H290 or GIF‐Q260 (Olympus Co., Tokyo, Japan). Vital signs (blood pressure, heart rate, and respiration rate), SpO_2_, and MOAA/S scores were measured at 5‐min intervals. The sedation level was assessed at 5‐min intervals using the MOAA/S score; if the MOAA/S score was 5, a further dose of either remimazolam or midazolam was administered. In addition, if there were signs of awakening (MOAA/S score 5 or body movements) other than at the 5‐min intervals, a further dose of remimazolam or midazolam was administered after an interval of at least 2 min since the previous dose had elapsed.

### 2.5 Post‐Endoscopy

Vital signs (blood pressure, heart rate, and respiration rate), SpO_2_, and MOAA/S score were assessed at the end of the endoscopy and after 5, 10 min, and subsequently at 10‐min intervals until 60 min had elapsed. If the MOAA/S score was 5, the patients were assessed to determine if they were ambulatory. Patients were considered ambulatory if they could walk along a 5‐m straight line without staggering.

### 2.6 Definition of Adverse Events

Adverse events associated with sedatives were investigated from the time of drug administration until the patient left the endoscopy room. The severity of adverse events was determined on the basis of the Common Terminology Criteria for Adverse Events, and changes in vital signs requiring medical intervention of Grade 2 or higher were treated as adverse events associated with sedatives. Medical interventions were performed in the midazolam and remimazolam groups according to the protocol of the REM‐IICT JP01 study. If systolic blood pressure was ≤ 90 mmHg on two consecutive occasions, hypotension was confirmed, and fluid loading or ephedrine was administered. If the SpO_2_ was < 94%, hypoxemia was confirmed; in that case, appropriate verbal encouragement was provided, and 2–5 L of oxygen was administered. A respiratory rate below 10 breaths per minute was determined to be respiratory depression, and appropriate verbal encouragement was given, and if the respiratory rate fell below 5 breaths per minute, airway management (head tilt‐chin lift maneuver) was performed. If breathing did not improve despite airway management, manual ventilation was performed using a bag valve mask. Flumazenil was administered intravenously if emergency antagonism of sedation from the trial drug was needed.

### 2.7 Outcomes

The primary outcome was the time from the end of the upper gastrointestinal endoscopy until discharge (meeting all the criteria of SpO_2_ > 92%, blood pressure > 90 mmHg, awakened, and ambulatory). The secondary outcomes were the time from the end of the upper gastrointestinal endoscopy until awakening (MOAA/S score 5), the sedative dose administered at the start of the procedure, number of additional doses, total dose administered, endoscopy time, and adverse events. We also performed propensity score matching to conduct a comparative investigation between similar groups.

### 2.8 Statistical Analysis

Continuous variables are expressed as median and interquartile range (IQR). The Mann–Whitney *U* test was used for comparisons between the groups. The Fisher exact test was used to compare categorical variables. In all cases, *p* < 0.05 was considered indicative of significant findings. Because this was a retrospective study and patient characteristics varied, we used propensity score matching to conduct a comparative investigation of the efficacy and safety in the two groups. The two groups were matched at a 1:1 ratio with adjustment for four covariates (age [years], sex [M/F], BMI, and American Society of Anesthesiologists [ASA] classification) to minimize inherent bias. These categories may affect the efficacy of sedatives and were set as covariates for propensity score matching. Propensity score matching was conducted using logistic regression analysis. This model yielded a *C* statistic of 0.80, indicating a preferable ability for comparison between two groups. The caliper value for propensity score matching was set to 0.20. JMP (version 13.0.0, SAS Institute, Cary, NC, USA) was used for all statistical analysis.

## 3 Results

### 3.1 Pre‐Propensity Score Matching

In the remimazolam and midazolam groups, all patients achieved a MOAA/S score of ≤ 4 before upper gastrointestinal endoscopy, and sedation was successful.

Table 2 shows the patient characteristics of both groups before propensity score matching. Statistically significant differences were observed in age, BMI, and ASA classification.

**TABLE 2:** Comparison of patient characteristics between the two groups before propensity score matching.

	Group M (*N* = 199)	Group R (*N* = 36)	*p*
Age (years)	64.0 (52.0–73.0)	50.0 (43.0–55.0)	< 0.01
Sex (*n*)			
M/F	109 (54.8%)/90 (45.2%)	14 (38.9%)/22 (61.1%)	0.10
Height (cm)	163.0 (156.8–169.0)	160.1 (156.1–171.0)	0.61
Weight (kg)	60.5 (52.5–70.0)	55.0 (50.2–63.6)	0.05
BMI (kg/m^2^)	23.1 (20.5–25.2)	21.1 (20.2–22.9)	0.01
ASA class (*n*)			
I	50 (25.1%)	26 (72.2%)	< 0.01
II	114 (57.3%)	9 (25.0%)	
III	35 (17.6%)	1 (2.8%)	
IV	0 (0%)	0 (0%)	
V	0 (0%)	0 (0%)	

*Note:* Data are presented as median (interquartile range) or number (percentage).

Abbreviations: ASA, American Society of Anesthesiologists classification; BMI, body mass index; F, female; Group M, midazolam group; Group R, remimazolam group; M, male.

Table 3 shows the sedation efficacy in both groups. The median time from the end of the procedure until awakening (MOAA/S score 5) was 33.0 min (26.0–42.0 min) in Group M and 0 min (0–5.0 min) in Group R, and the median time from the end of the upper gastrointestinal endoscopy until discharge was 42.0 min (37.0–51.0 min) in Group M and 5.0 min (0–5.0 min) in Group R, both significantly shorter in Group R (*p* < 0.01).

**TABLE 3:** Comparison of the efficacy and safety of sedation between the two groups before propensity score matching.

	Group M (*N* = 199)	Group R (*N* = 36)	*p*
Pre‐procedure dose (mg)	2.0 (2.0–3.0)	3.0 (3.0–4.0)	
Additional dose (*n*)	0 (0–0)	1 (0–1)	
Total dose (mg)	2.5 (2.0–3.0)	4.0 (3.0–5.0)	
Endoscopy time (min)	5.0 (4.0–8.0)	6.0 (5.0–7.0)	0.82
Time from the end of the procedure until awakening (MOAA/S score 5) (min)	33.0 (26.0–42.0)	0 (0–5.0)	< 0.01
Time from the end of the upper gastrointestinal endoscopy until discharge (min)	42.0 (37.0–51.0)	5.0 (0–5.0)	< 0.01
Adverse events (*n*)			
Hypotension	0 (0%)	1 (2.8%)	0.15
Hypoxemia (SpO_2_ < 94%)	0 (0%)	1 (2.8%)	0.15
Respiratory depression	0 (0%)	0 (0%)	1.0

*Note:* Data are presented as median (interquartile range) or number (percentage).

Abbreviations: Group M, midazolam group; Group R, remimazolam group; MOAA/S, Modified Observer's Alertness/Sedation; SpO_2_, peripheral oxygen saturation.

The median dose administered before the start of the procedure was 2.0 mg (2.0–3.0 mg) in Group M and 3.0 mg (3.0–4.0 mg) in Group R, the median number of additional doses was 0 (0–0) in Group M and 1 (0–1) in Group R, and the median total dose administered was 2.5 mg (2.0–3.0) mg in Group M and 4.0 mg (3.0–5.0 mg) in Group R. The median endoscopy time was 5.0 min (4.0–8.0 min) in Group M and 6.0 min (5.0–7.0 min) in Group R. The adverse events reported were hypotension and hypoxemia in one patient in Group R.

### 3.2 Post‐Propensity Score Matching

Table 4 shows the patient characteristics of both groups after propensity score matching. Thirty‐four patients in both groups were matched. There were no statistically significant differences between the groups in age, sex, BMI, ASA class, or other background factors.

**TABLE 4:** Comparison of patient characteristics between the two groups after propensity score matching.

	Group M (*N* = 34)	Group R (*N* = 34)	*p*
Age (years)	54.0 (49.0–63.3)	50.0 (42.3–55.5)	0.06
Sex			
M/F (*n*)	14 (41.2%)/20 (58.8%)	14 (41.2%)/20 (58.8%)	1.0
Height (cm)	163.3 (158.8–169.5)	160.1 (156.3–171.4)	0.64
Weight (kg)	57.9 (50.6–65.8)	56.7 (50.3–63.9)	0.80
BMI (kg/m^2^)	21.7 (19.7–23.5)	21.1 (20.2–22.8)	1.0
ASA class (*n*)			
I	24 (70.6%)	24 (70.6%)	0.53
II	7 (20.6%)	9 (26.5%)	
III	3 (8.8%)	1 (2.9%)	
IV	0 (0.0%)	0 (0.0%)	
V	0 (0.0%)	0 (0.0%)	

*Note:* Data are presented as median (interquartile range) or number (percentage).

Abbreviations: ASA, American Society of Anesthesiologists classification; BMI, body mass index; F, female; Group M, midazolam group; Group R, remimazolam group; M, male.

Table 5 shows the results of the efficacy of sedation in the two groups after propensity score matching. The median time from the end of the procedure to awakening (MOAA/S score 5) was 27.0 min (23.0–40.5 min) in Group M and 0 min (0–5.0 min) in Group R, and the median time from the end of the upper gastrointestinal endoscopy until discharge was 39.0 min (35.0–52.5 min) in Group M and 5.0 min (0–5.0 min) in Group R, both significantly shorter in Group R (*p* < 0.01).

**TABLE 5:** Comparison of the efficacy and safety of sedation between the two groups after propensity score matching.

	Group M (*N* = 34)	Group R (*N* = 34)	*p*
Pre‐procedure dose (mg)	2.0 (2.0–3.0)	3.0 (3.0–4.0)	
Additional dose (*n*)	0 (0–0)	1 (0–1)	
Total dose (mg)	2.0 (2.0–3.0)	4.0 (3.0–5.0)	
Endoscopy time (min)	5.5 (4.0–10.3)	6.0 (5.0–7.0)	1.0
Time from the end of the procedure until awakening (MOAA/S score 5) (min)	27.0 (23.0–40.5)	0 (0–5.0)	< 0.01
Time from the end of the upper gastrointestinal endoscopy until discharge (min)	39.0 (35.0–52.5)	5.0 (0–5.0)	< 0.01
Adverse events (*n*)			
Hypotension	0 (0%)	1 (2.9%)	1.0
Hypoxemia (SpO_2_ < 94%)	0 (0%)	1 (2.9%)	1.0
Respiratory depression	0 (0%)	0 (0%)	1.0

*Note:* Data are presented as median (interquartile range) or number (percentage).

Abbreviations: Group M, midazolam group; Group R, remimazolam group; MOAA/S, Modified Observer's Alertness/Sedation; SpO_2_, peripheral oxygen saturation.

The median dose administered before the start of the procedure was 2.0 mg (2.0–3.0 mg) in Group M and 3.0 mg (3.0–4.0 mg) in Group R, the median number of additional doses was 0 (0–0) in Group M and 1 (0–1) in Group R, and the median total dose administered was 2.0 mg (2.0–3.0 mg) in Group M and 4.0 mg (3.0–5.0 mg) in Group R. The median endoscopy time was 5.5 min (4.0–10.3 min) in Group M and 6.0 min (5.0–7.0 min) in Group R.

## 4 Discussion

To our knowledge, this is the first study to compare upper gastrointestinal endoscopy under sedation with remimazolam and midazolam in Japanese patients. The time to awakening and the time until the patient was able to leave the endoscopy room were significantly shorter for remimazolam than for midazolam. Almost no adverse events were noted for either remimazolam or midazolam, indicating that they are safe for use. The half‐life of remimazolam is 45 min, which is much shorter than the 4‐h half‐life of midazolam and remimazolam, and its use should shorten the time until the patient can leave the endoscopy room after the procedure.

Only a few studies have compared the use of midazolam and remimazolam during upper gastrointestinal endoscopy. One study in the United States in which upper gastrointestinal endoscopy was conducted on 20 patients each using sedation with remimazolam (0.10, 0.15, or 0.20 mg/kg) or midazolam (0.075 mg/kg) found that the sedation success rates were 32.0%, 56.0%, 64.0%, and 44.0% and the times to awakening were 6.8, 9.0, 9.9, and 11.5 min, respectively. Although this study had a small sample size, endoscopy using remimazolam (0.20 mg/kg) had a higher sedation success rate and earlier awakening than that using midazolam (0.075 mg/dL) [15].

Another study of endoscopic treatment of intensive care unit (ICU) patients under sedation with either remimazolam or conventional sedation with midazolam or propofol found no significant difference in treatment time (35.89 ± 13.37 and 44.51 ± 21.68 min, respectively, *p* = 0.133), duration of ICU stay (5.40 ± 2.93 and 4.63 ± 3.31 days, respectively, *p* = 0.072), or the incidence of adverse events (48.61% and 51.38%, respectively, *p* = 0.056). However, the use of remimazolam decreased medical costs [21].

A meta‐analysis that compared the efficacy and safety of remimazolam and midazolam in an endoscopy reported that remimazolam had a higher sedation success rate, more rapid awakening, and a lower incidence of hypotension and other complications than midazolam [22]. However, although the meta‐analysis included three randomized controlled trials (RCTs), two of the RCTs were on colonoscopy, and no meta‐analysis has compared remimazolam and midazolam in upper gastrointestinal endoscopy. These were also compared with sedation under concomitant sedatives or with propofol as a rescue dose, which may have affected the effectiveness of sedation. This is an important study on upper gastrointestinal endoscopy under sedation with either remimazolam or midazolam alone.

Remimazolam can also be used safely in pediatric endoscopy [23] and does not increase the risk of hepatic encephalopathy in patients with poor hepatic function compared with midazolam [24]. This suggests that remimazolam is a promising sedative that can be used during endoscopic procedures in a wide range of patients. Remimazolam for sedation during upper gastrointestinal endoscopy may shorten the time until recovery and reduce the number of staff required in the recovery room. In addition, the shortened recovery time is expected to increase the number of endoscopic examinations per day and reduce the waiting period for examinations.

This study has some limitations. First, because it was a retrospective single‐center study, the remimazolam group consisted of clinical trial participants, and their patient characteristics differed from those of the midazolam group. Therefore, we adjusted for patient characteristics by propensity score matching. Selection bias may not be completely eliminated. Therefore, further randomized prospective trials at multiple centers are required. Second, our sample size was small, and the study was restricted to Japanese participants. Thus, our results cannot be generalized to all races. Further studies with more patients are required.

In conclusion, remimazolam can enhance clinical efficiency in endoscopy units by decreasing recovery times while simultaneously minimizing the incidence of adverse events. This could make it a safer and more efficient sedative option for gastrointestinal procedures, ultimately improving patient outcomes.

## Ethics Statement

The main study (REM‐IICT JP01) protocol was approved by the Institutional Review Board of each participating institution and was registered in the Japan Registry of Clinical Trails (jRCT2031200360). The current study was a post hoc analysis of a study (REM‐IICT JP01) and was approved by the Institutional Review Board of Nihon University Hospital. This study was conducted in compliance with the principles of the Declaration of Helsinki.

## Consent

Written informed consent was obtained from all patients who underwent endoscopy.

## Conflicts of Interest

The authors declare no conflicts of interest.

## Data Availability

The data that support the findings of this study are not publicly available owing to their containing information that could compromise the privacy of research participants but are available from corresponding author upon reasonable request.
